# Deficiency of Thioredoxin Binding Protein-2 (TBP-2) Enhances TGF-β Signaling and Promotes Epithelial to Mesenchymal Transition

**DOI:** 10.1371/journal.pone.0039900

**Published:** 2012-06-29

**Authors:** So Masaki, Hiroshi Masutani, Eiji Yoshihara, Junji Yodoi

**Affiliations:** 1 Laboratory of Infection and Prevention, Department of Biological Responses, Institute for Virus Research, Kyoto University, Kyoto, Japan; 2 Department of Bioinspired Sciences, Center for Cell Signaling Research, Ewha Womans University, Seoul, South Korea; Leiden University Medical Center, The Netherlands

## Abstract

**Background:**

Transforming growth factor beta (TGF-β) has critical roles in regulating cell growth, differentiation, apoptosis, invasion and epithelial-mesenchymal transition (EMT) of various cancer cells. TGF-β-induced EMT is an important step during carcinoma progression to invasion state. Thioredoxin binding protein-2 (TBP-2, also called Txnip or VDUP1) is downregulated in various types of human cancer, and its deficiency results in the earlier onset of cancer. However, it remains unclear how TBP-2 suppresses the invasion and metastasis of cancer.

**Principal Findings:**

In this study, we demonstrated that TBP-2 deficiency increases the transcriptional activity in response to TGF-β and also enhances TGF-β-induced Smad2 phosphorylation levels. Knockdown of TBP-2 augmented the TGF-β-responsive expression of Snail and Slug, transcriptional factors related to TGF-β-mediated induction of EMT, and promoted TGF-β-induced spindle-like morphology consistent with the depletion of E-Cadherin in A549 cells.

**Conclusions/Significance:**

Our results indicate that TBP-2 deficiency enhances TGF-β signaling and promotes TGF-β-induced EMT. The control of TGF-β-induced EMT is critical for the inhibition of the invasion and metastasis. Thus TBP-2, as a novel regulatory molecule of TGF-β signaling, is likely to be a prognostic indicator or a potential therapeutic target for preventing tumor progression.

## Introduction

Transforming growth factor-β (TGF-β) has dual functions in cancer [1]. TGF-β acts as a tumor suppressor in the early stage of tumor development, and contradictorily, promotes the invasion and metastasis of tumor cells in the late stage. Recently, many studies have shown that TGF-β promotes cancer progression by inducing Epithelial-mesenchymal transition (EMT), which is a crucial process to acquire the ability to execute the invasion-metastasis steps of cancer [2], [3]. TGF-β induces the expression of several transcription factors driven to EMT [4], including Snail/SNAI1 [5] and Slug/SNAI2 [6], which act directly or indirectly as a repressor of E-Cadherin. The loss of E-Cadherin is a fundamental event in EMT [7], [8].

Thioredoxin binding protein-2 (TBP-2), also known as thiredoxin interacting protein (Txnip) [9] or Vitamin D3 upregulated protein 1 (VDUP1) [10], has been identified as a negative regulator of thioredoxin (TRX) [11] and is mainly localized in nucleus [12]. TBP-2 is a member of α-arrestin protein family, and contains two PPxY motifs, which are known to interact with WW domain-containing proteins including Nedd4 family of E3 ubiquitin ligases [13], [14]. TBP-2 has a variety of biological functions in cell proliferation [15], cell apoptosis [16], immune response [17], [18], [19], glucose and lipid metabolism [9], [20], [21], [22], [23], [24].

There is the growing evidence that TBP-2 plays as a suppressor of cancer. TBP-2 is downregulated in various human cancer cells [25], [26]. TBP-2 overexpression inhibits proliferation via cell cycle arrest [12], [27], [28], [29] and promotes apoptosis [30]. In human T cell lymphocyte virus type 1 (HTLV-I)- infected T cells, TBP-2 regulates cell growth and its expression is associated with responsiveness to IL-2-dependent growth [31], and plays a key role in glucocorticoid-induced cell death [32]. *In vivo* studies, TBP-2 overexpression suppressed tumor growth and metastasis of the transplanted tumor. Point mutation or knock out of TBP-2 gene in mice show the higher incidence of hepatocellular carcinoma [33], [34]. TBP-2 knock out mice also shows the earlier onset of N-butyl-N- (4-hydroxybutyl) nitrosamine (BBN)-induced bladder carcinoma [35].

**Figure 1:**
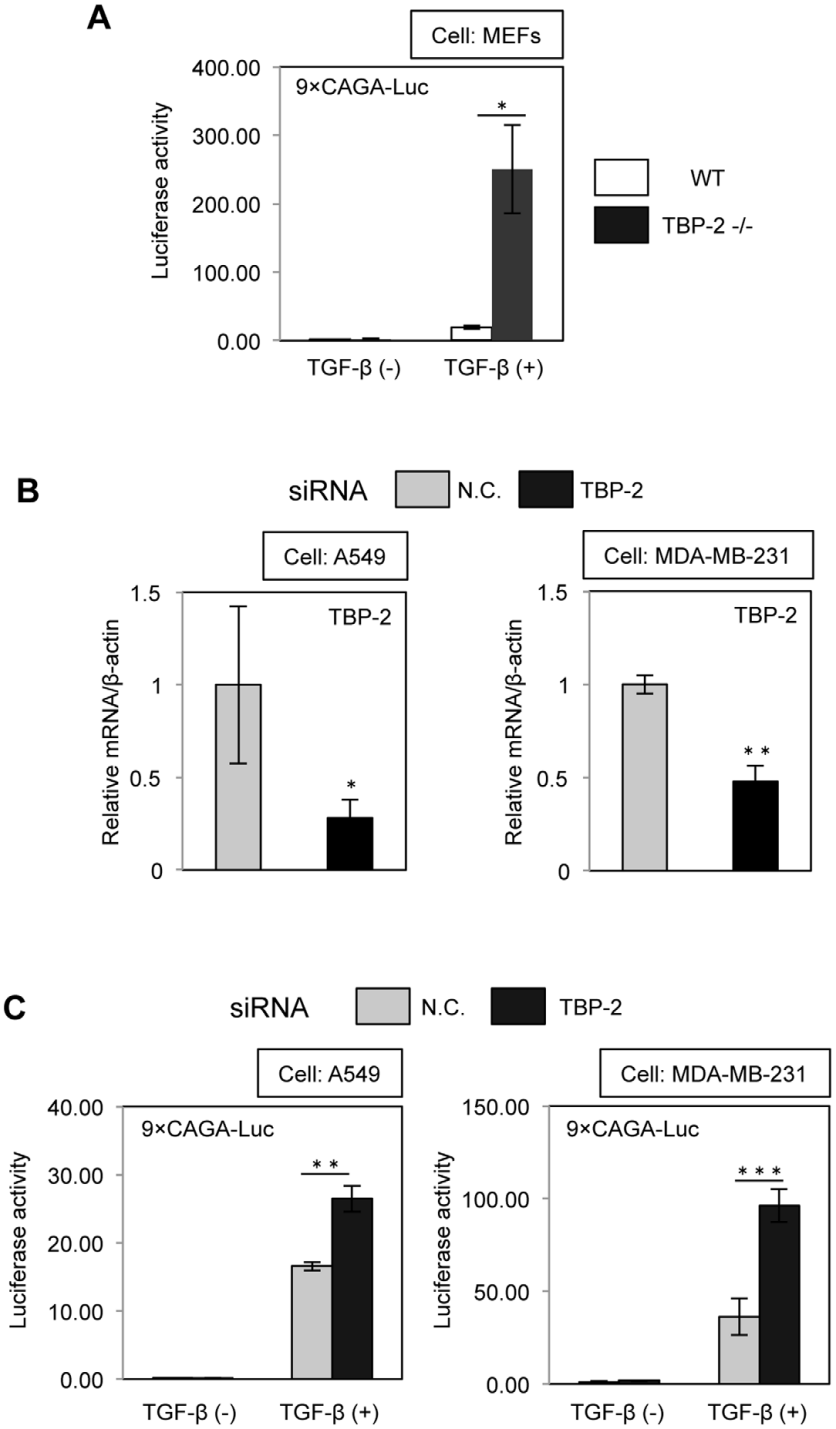
Deficiency of TBP-2 enhances the transcriptional activity of TGF-β signaling. (A) Effect of TBP-2 deficiency on the transcriptional activity of TGF-β was examined using 9×CAGA-MLP-Luc, TGF-β responsive luciferase reporter, in WT (Wild Type: TBP-2^+/+^) and TBP-2^−/−^ MEFs with or without TGF-β (0.5 ng/ml). (B) The efficiency of TBP-2 knockdown by TBP-2 siRNA and negative control (N.C.) in A549 and MDA-MB-231 cells was determined by quantitative real-time PCR at 36 hours after transfection. (C) Effect of TBP-2 knockdown on the transcriptional activity of TGF-β was examined using 9×CAGA-MLP-Luc in A549 and MDA-MB-231 cells with or without TGF-β. N.C. means negative control. The error bars show mean ± SD. ^*^ *P*<0.05, ^**^ *P*<0.01, ^***^ *P*<0.001, versus control (*t*-test).

These results collectively support that TBP-2 deficiency contributes to the progression and metastasis of cancer, however, detail mechanisms of TBP-2 in this process has not been sufficiently elucidated. In the late stage of cancer cells, TBP-2 expression is downregulated and TGF-β elicits cancer malignancy driving EMT. This correlation provides the hypothesis that TBP-2 regulates TGF-β-associated cancer development in the late stage.

In the present study, we examined the role of TBP-2 in TGF-β signaling. TBP-2 deficiency increased TGF-β signaling by enhancing Smad2 phosphorylation levels, and upregulated TGF-β-induced expression of Snail or Slug, resulting in acceleration of TGF-β-driven EMT. These findings show a novel function of TBP-2, as a regulator of TGF-β signaling, and provide new insights to the mechanisms of TGF-β-induced EMT.

## Results

### TBP-2 Deficiency Enhances Transcriptional Activity of TGF-β Signaling

To investigate the function of TBP-2 in TGF-β signaling, we performed promoter assay using 9×CAGA-Luc (TGF-β-responsive promoter-reporter), which is the most frequently used reporter system for TGF-β/Smad signal transduction, in WT (Wild Type: TBP-2^+/+^) mouse embryonic fibroblasts (MEFs) and TBP-2^−/−^ MEFs. The results showed that transcriptional activity in response to TGF-β is enhanced in TBP-2^−/−^ MEFs compared with WT MEFs (Fig. 1A). The efficiency of TBP-2 knockdown in A549 and MDA-MB-231 cells was confirmed by real-time RT-PCR (Fig. 1B). All experiments with TBP-2 siRNA were done according to the same protocol. Knockdown of TBP-2 also resulted in enhancing TGF-β-induced transcriptional activity in A549, MDA-MB-231 (Fig. 1C) and 253J (data not shown) cell lines.

### TBP-2 Deficiency Increases the mRNA Expression of TGF-β-targeted Genes

**Figure 2:**
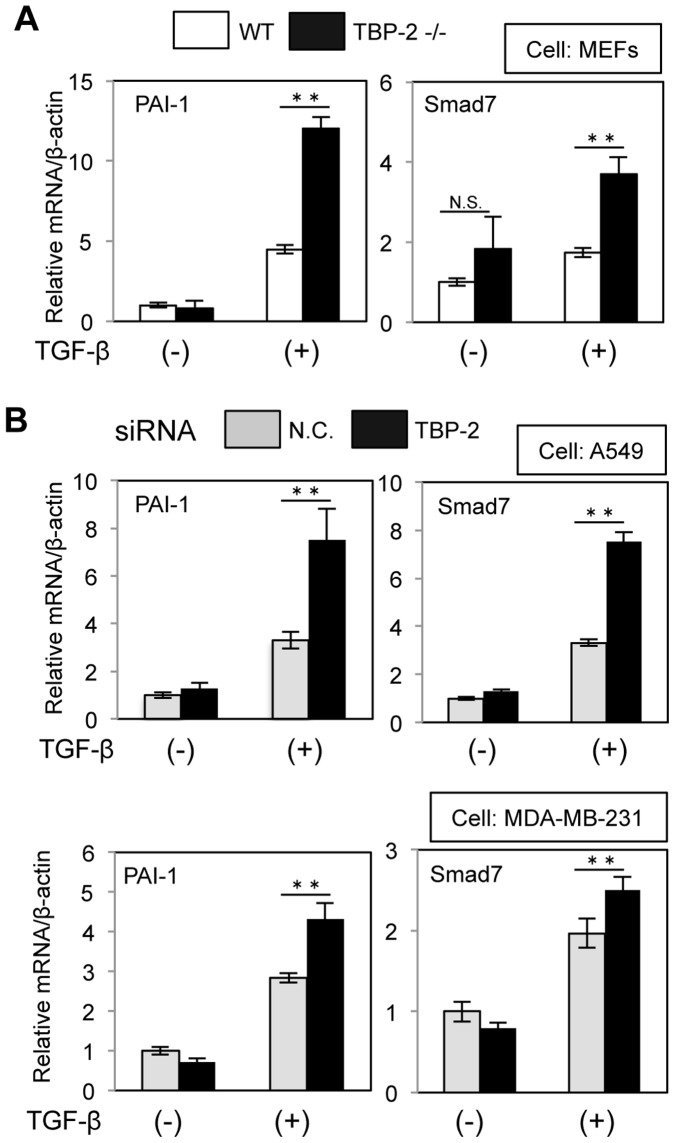
Deficiency of TBP-2 upregulates mRNA of TGF-β targeted genes. (A) TGF-β-induced mRNA expression of PAI-1 or Smad7, TGF-β targeted genes, in WT and TBP-2^−/−^ MEFs was determined by quantitative real-time PCR. MEFs were cultured in the presence or absence of TGF-β (0.5 ng/ml) for 8 hours. (B) The effects of TBP-2 knockdown for TGF-β-induced mRNA expression of PAI-1 or Smad7 in A549 cells and MDA-MB-231 cells were determined by quantitative real-time PCR. A549 cells and MDA-MB-231 cells were cultured in the presence or absence of TGF-β (2.5 ng/ml for 6 hours and 1 ng/ml for 12 hours, respectively). N.C. means negative control. The error bars show mean ± SD. **P<0.01, N.S.: not significant.

To further examine that TBP-2 regulates the expression of TGF-β-target genes, plasminogen activator inhibitor (PAI)-1 and Smad7, well known TGF-β-targeted genes, were quantified by real-time RT-PCR. TGF-β-mediated induction of PAI-1 and Smad7 is increased in TBP-2^−/−^ MEFs (Fig. 2A), as well as A549 and MDA-MB-231 cells under the condition of TBP-2 knockdown (Fig. 2B).

### TBP-2 Deficiency Increases TGF-β-mediated Phosphorylation of Smad2

**Figure 3:**
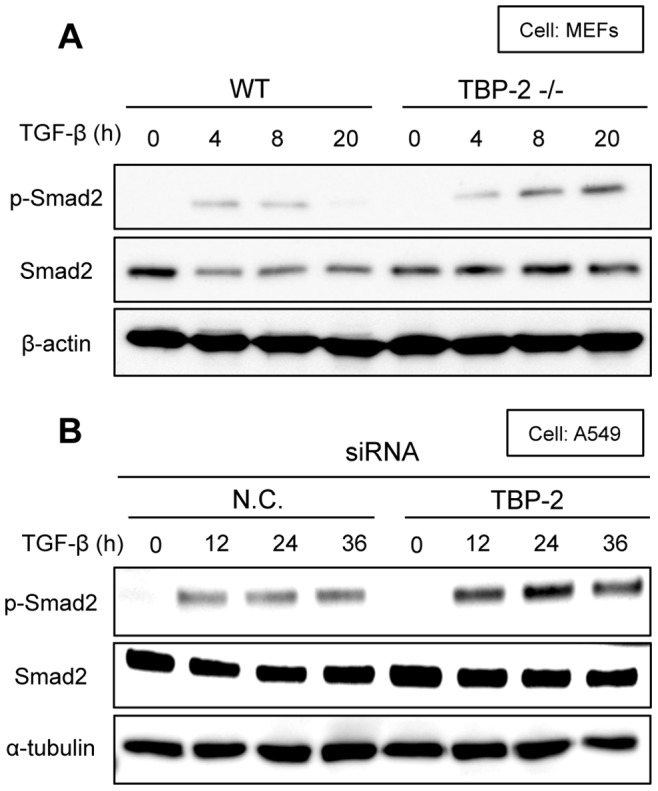
Deficiency of TBP-2 maintains the higher phosphorylation level of Smad2. (A) WT and TBP-2^−/−^ MEFs cells were stimulated with TGF-β (0.5 ng/ml) for the indicated times. p-Smad2, Smad2 and β-actin were analyzed by Western blot. (B) A549 cells under the condition of TBP-2 knockdown or not were stimulated with TGF-β (2.5 ng/ml) for the indicated times. p-Smad2, Smad2 and α-tubulin were analyzed by Western blot. N.C. means negative control.

Next, we analyzed the level of TGF-β-mediated phosphorylation of Smad2 in WT and TBP-2^−/−^ MEFs by the western blot analyses. The phospho-Smad2 protein level was declined at 20 hour-TGF-β stimulation in WT MEFs, but was continuously elevated in TBP-2^−/−^ MEFs (Fig. 3A). Similarly, phospho-Smad2 levels were enhanced with TGF-β stimulation for 12, 24 and 36 hours in TBP-2 knockdown-A549 cells (Fig. 3B). In addition, total Smad2 protein levels went down for 4 hours, responding to TGF-β stimulation, but were unchanged between 4 to 20 hours in WT MEFs, whereas no significant differences from 0 to 20 hours with TGF-β stimulation in TBP-2^−/−^ MEFs (Fig. 3A).

### TBP-2 Deficiency Enhances the Induction of Snail and Slug by TGF-β

**Figure 4:**
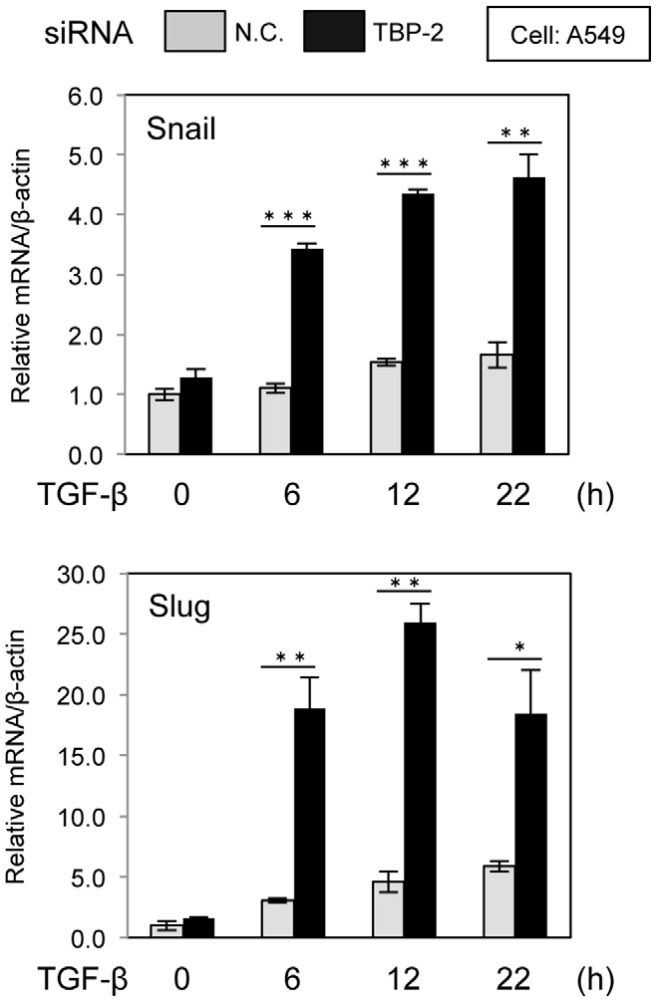
Knockdown of TBP-2 promotes Snail and Slug induction by TGF-β. Induction of Snail (A) or Slug (B) transcription was examined in A549 cells under the condition of TBP-2 knockdown (black bars) or not (gray bars) cultured with TGF-β (2.5 ng/ml) for the indicated times. Snail or Slug mRNA were determined by quantitative real-time PCR. N.C. means negative control. The error bars show mean ± SD. *P<0.05, **P<0.01, ***P<0.001, versus control (*t*-test).

TGF-β induces the expression of transcriptional factors involved in EMT, including Snail and Slug. As the induction of Snail or Slug is a crucial step for EMT, the effect of TBP-2 knockdown on the induction of Snail and Slug by TGF-β was examined with real-time RT-PCR. The results showed that the TGF-β-responsive expression of Snail and Slug was enhanced with TGF-β stimulation for 6, 12 and 22 hours in A549 cells under the condition of TBP-2 knockdown (Fig. 4).

### TBP-2 Deficiency Promotes TGF-β-induced EMT

**Figure 5:**
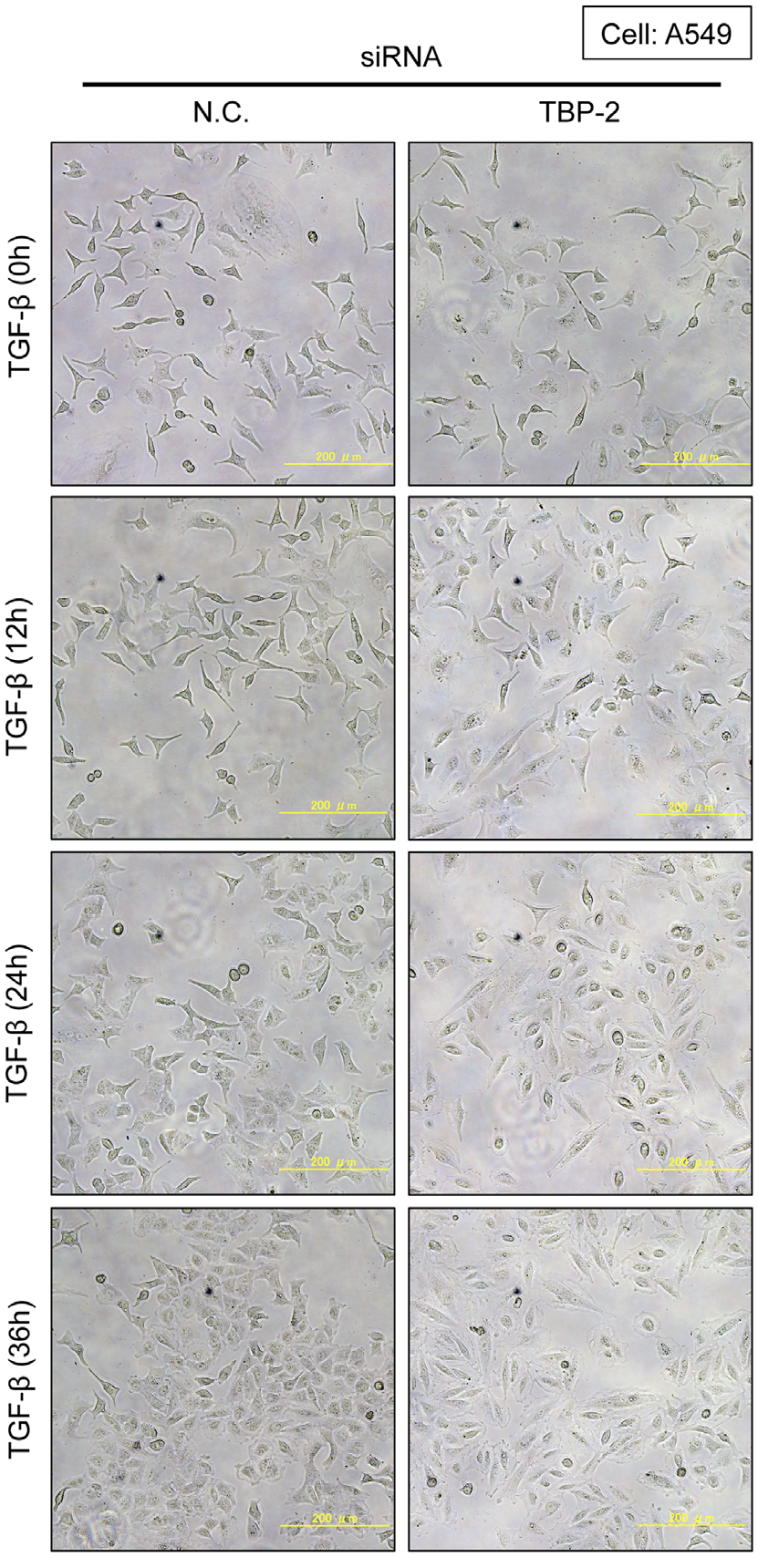
Knockdown of TBP-2 accelerates TGF-β-induced cell morphological changes. A549 cells transfected with TBP-2-targeting siRNA (TBP-2) or negative control siRNA (N.C.) were cultured in the presence of TGF-β (2.5 ng/ml) for 0, 12, 24 and 36 hours. Photos were taken at the indicated times.

**Figure 6:**
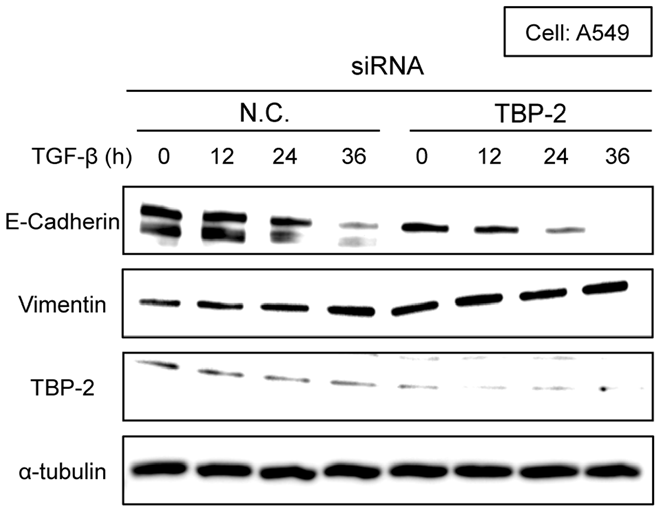
Knockdown of TBP-2 accelerates TGF-β-driven E-Cadherin degradation. A549 cells transfected with TBP-2-targeting siRNA (TBP-2) or negative control siRNA (N.C.) were treated with TGF-β (2.5 ng/ml) for 0, 12, 24 and 36 hours. E-Cadherin, Vimentin, TBP-2 and α-tubulin were analyzed by Western blot.

Then, we evaluated the effects of TBP-2 knockdown in TGF-β-induced EMT. Knockdown of TBP-2 promoted TGF-β-induced morphological changes in A549 (Fig. 5) and 253J cells (data not shown). In the presence of 2.5 ng/ml TGF-β for 24 or 36 hours, TGF-β-driven spindle-like morphology was significantly observed in TBP-2 knockdown-A549 cells. To quantify the morphological changes, we measured the length of the longest diagonal line of each cell. TBP-2 knockdown-cells with TGF-β stimulation significantly lengthened more than control cells (Fig. S1). Consistently, the depletion of E-Cadherin, an epithelial marker, was quickened, and similarly the induction of vimentin, a mesenchymal marker, was elevated in TBP-2 knockdown-A549 cells (Fig. 6). These results indicate that TBP-2 deficiency accelerates the TGF-β-driven EMT phenotype.

## Discussion

In this study, we demonstrated that deficiency of TBP-2 increases TGF-β-responsive transcriptional activity and upregulates Smad2 phosphorylation levels, resulting in the acceleration of TGF-β-induced EMT.

TBP-2 deficiency contributes to upregulate transcriptional activities for several stimuli or ligands. We or other groups reported that peroxisome proliferator activated receptor (PPAR) or insulin target genes are upregulated in TBP-2^−/−^ mice, and that TBP-2 negatively regulates PPAR transcriptional activity *in vitro* [23]. TBP-2 deficiency might maintain the level of transcriptional activities with the imperfection of biological feedback.

TBP-2 deficiency also results in the enhancement of phosphorylation of signal transducers. Regarding the relationship between TBP-2 and cell signaling, it was reported that phosphorylation of ERK is enhanced in TBP-2-KO mice [36] bladders during BBN-induced bladder carcinogenesis [35]. Our previous study showed that TBP-2 is a negative regulator of TRX [11], and other group reported that overexpression of TRX elevates the ERK1/2 phosphorylation levels [37]. These reports suggest that TBP-2 deficiency facilitates TRX activity, resulting in enhancement of the phosphorylation levels of signal transducer, such as ERK1/2. However, TBP-2 deficiency did not change the protein levels of TRX in the presence or absence of TGF-β (data not shown), so that TRX might not be related to the regulation of TGF-β by TBP-2.

The re-expression of TBP-2 using expression vector in TBP-2^−/−^ MEFs failed to rescue the knock out effects of TBP-2 on the CAGA promoter. We also performed the experiments on the gain-of-function of TBP-2 using expression vector in A549 and MDA-MB-231 cell lines. The results unexpectedly showed that the overexpression of TBP-2 did not lead to the opposite of the loss-of-function results (data not shown). These results might be caused by the difficulty in controlling the expression level of TBP-2 within the physiological range. Since TBP-2 is a multifunctional protein targeting several molecules, the superabundant expression of TBP-2 might cause unexpected effects, which should be dissected in our future study.

It has been also reported that TBP-2 deficiency promotes TNF-α-induced NF-κB activity [34], that TBP-2 inhibits mTOR activity by binding REDD1 protein [38], and that TBP-2 deficiency enhances the phosphorylation of Akt in response to insulin [16], [24]. The present study shows that TBP-2 deficiency enhances TGF-β-mediated Smad2 phosphorylation level. These findings suggest that TBP-2 act as a crucial feedback regulator for various biological responses. TBP-2 might be essential for protein phosphatases or protein degradation systems.

TBP-2 deficiency enhanced TGF-β signaling and upregulated Smad7 expression (Fig. 1 and 2). Smad7, one of inhibitory Smads, plays an essential role in the negative feedback regulation of TGF-β signaling [39], however, TBP-2 deficiency enhanced TGF-β-mediated Smad2 phosphorylation (Fig. 3) irrespective of increasing Smad7 expression. In the negative feedback of TGF-β signaling, Smad7 requires to bind to Smad ubiquitin regulatory factor 2 (Smurf2), HECT type E3 ligases containing WW domain [39], [40]. Smad7-Smurf2 complex binds to the activated TGF-β receptors, and induces their degradation [41], [42]. In addition, Smurf2 also decreases the protein levels of Smad2 in response to TGF-β stimulation. Our results showed that total Smad2 protein levels went down for 4 hours, responding to TGF-β stimulation in WT MEFs, but no significant differences in TBP-2^−/−^ MEFs. TBP-2 contains two PPxY motifs, which are reported to interact with WW domain. TBP-2 interacts with Smurf2 in co-immnoprecipitation assay (data not shown), providing the hypothesis that TBP-2 is required for functions of Smurf2 in the negative feedback of TGF-β signaling. The significance of TBP-2-Smurf2 interaction has been entirely unclear and will be examined in detail.

In conclusion, we demonstrated that TBP-2 deficiency enhances Smad2 phosphorylation level, resulting in acceleration of TGF-β-driven EMT. Our findings show a novel mechanisms of cancer suppression associated with TBP-2 and provide new insights into TGF-β-mediated EMT. TBP-2 is likely to be a prognosis indicator by monitoring TBP-2 expression in tumor, and a potential therapeutic target in the inhibition of EMT.

## Materials and Methods

### Reagents and Antibodies

TGF-β1 was purchased from R&D systems. Stealth small interfering RNA (siRNA) for TBP-2 (UCAAUUCGAGCAGAGACAGACACCC) and a negative control were purchased from Invitrogen. The antibodies used were as follows: anti-phospho-Smad2 (Ser465/467) (138D4) and anti-Smad2 (L16D3) antibodies were purchased from Cell Signaling. Anti-Txnip antibody and Anti-Vimentin were from MBL. Anti-E-Cadherin antibody was from Transduction Laboratories. Anti-β-actin antibody was from Santa Cruz. Anti-α-tubulin antibody was from Sigma.

### Cell Culture

Primary wild-type and TBP-2^−/−^ mouse embryonic fibroblasts (MEFs) were generated as previously described [24]. Human lung adenocarcinoma cell line A549 was obtained from Health Science Research Bank. Human breast cancer cell line MDA-MB-231 was from DS Pharma Biomedical. MEFs, A549 and MDA-MB-231 cells were cultured in Dulbecco’s modified Eagle’s medium (DMEM) with 10% fetal bovine serum (FBS), 1% penicillin/streptomycin antibiotics, and 2 mM L-glutamine. The culture was maintained at 37°C with 5% CO_2_.

### RNA Interference

All knockdown assay using siRNAs were performed with Lopofectamine 2000 (Invitrogen) according to the manufacturer’s instruction. The cells were used after 36 hours from transfection.

### Transient Transfection and Luciferase Reporter Assay

Cells were transiently transfected with pGL3 9×CAGA-MLP-Luc and pRL-TK (Promega) using TransIT-LT1 (Takara) according to the manufacturer’s instruction. pRL-TK was used as a control of the efficiency of transfection. At the same time of transfection, cells were under the condition of serum deprivation. After 20 hours of transfection, cells were stimulated with TGF-β for 20 hours. Luciferase activity was measured with the Dual-Luciferase reporter system (Promega).

### RNA Isolation, RT-PCR and Real-time Quantitative PCR

Total RNAs were extracted using TRIzol (Invitrogen), and were reverse-transcribed using High-Capacity cDNA Reverse Transcription Kits (Applied Biosystems) according to the manufacturer’s instruction. Real-time PCR was performed with Power STBR Green PCR Master Mix (Applied Biosystems), using β-actin as an internal control for normalization. Fluorescent detection and data analyses were performed using ABI 7500 Sequence Detection System (Applied Biosystems). Primers for PCR analyses were listed in Table S1.

### Immunoblotting Analysis

For western blotting, the cells were lysed in CelLytic M Cell Lysis Reagent (Sigma-Aldrich) containing a protease inhibitor cocktail (Roche) and phosphatase inhibitor (Nacalai Tesque). The lysate were boiled with Laemmli Smaple Buffer (BIO-RAD) at 95°C for 3 minutes. The samples were subjected to SDS-PAGE, transferred to PVDF membranes, and incubated with primary antibodies. The membranes were washed and incubated with horseradish peroxidase-conjugated secondary anti-mouse- or anti-rabbit-immunoglobulin G (GE Lifesciences). Finally, chemiluminescence was detected using Chemi-Lumi One L kit (Nacalai Tesque), and luminescence images were analyzed by LAS 3000 or LAS 4000 (GE Lifesciences).

## Supporting Information

Figure S1 **The length of the longest diagonal line of TBP-2 siRNA-A549 and control siRNA-A549 cells in the presence or absence of TGF-β (2.5 ng/ml for 36 hours).** The length of each cell was calculated from expanded photos (200 cells).
(TIF)

Table S1 Primer sequences for real-time PCR analyses.
(TIFF)
