# Supplementary figures and images for: Deficiency of Thioredoxin Binding Protein-2 (TBP-2) Enhances TGF-β Signaling and Promotes Epithelial to Mesenchymal Transition
Source: PLoS One. 2012 Jun 29;7(6):e39900. doi: 10.1371/journal.pone.0039900 (PMC3387201; doi:10.1371/journal.pone.0039900)

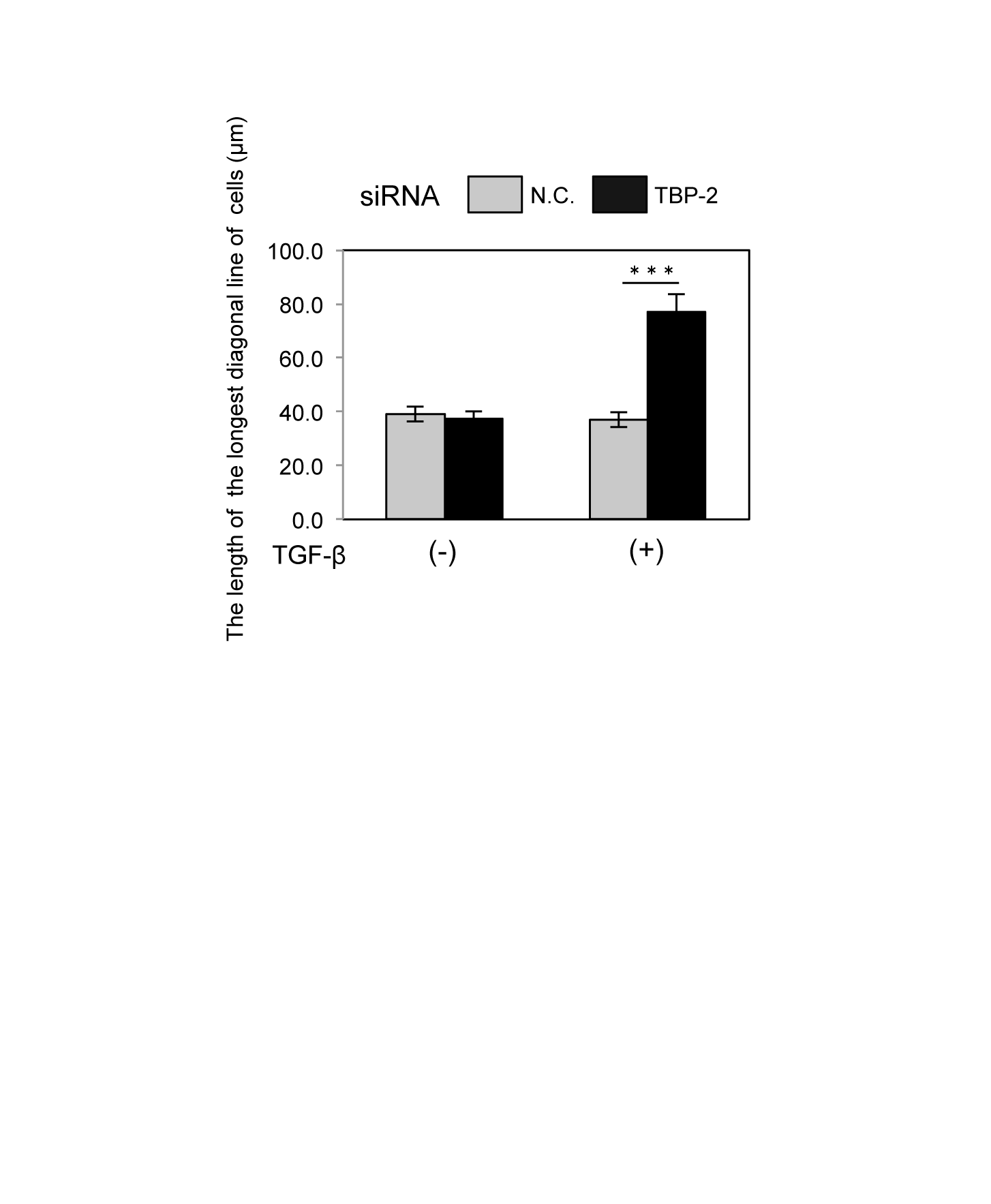

Supplement: Figure S1 — The length of the longest diagonal line of TBP-2 siRNA-A549 and control siRNA-A549 cells in the presence or absence of TGF-β (2.5 ng/ml for 36 hours). The length of each cell was calculated from expanded photos (200 cells). (TIF) [file pone.0039900.s001.tif]

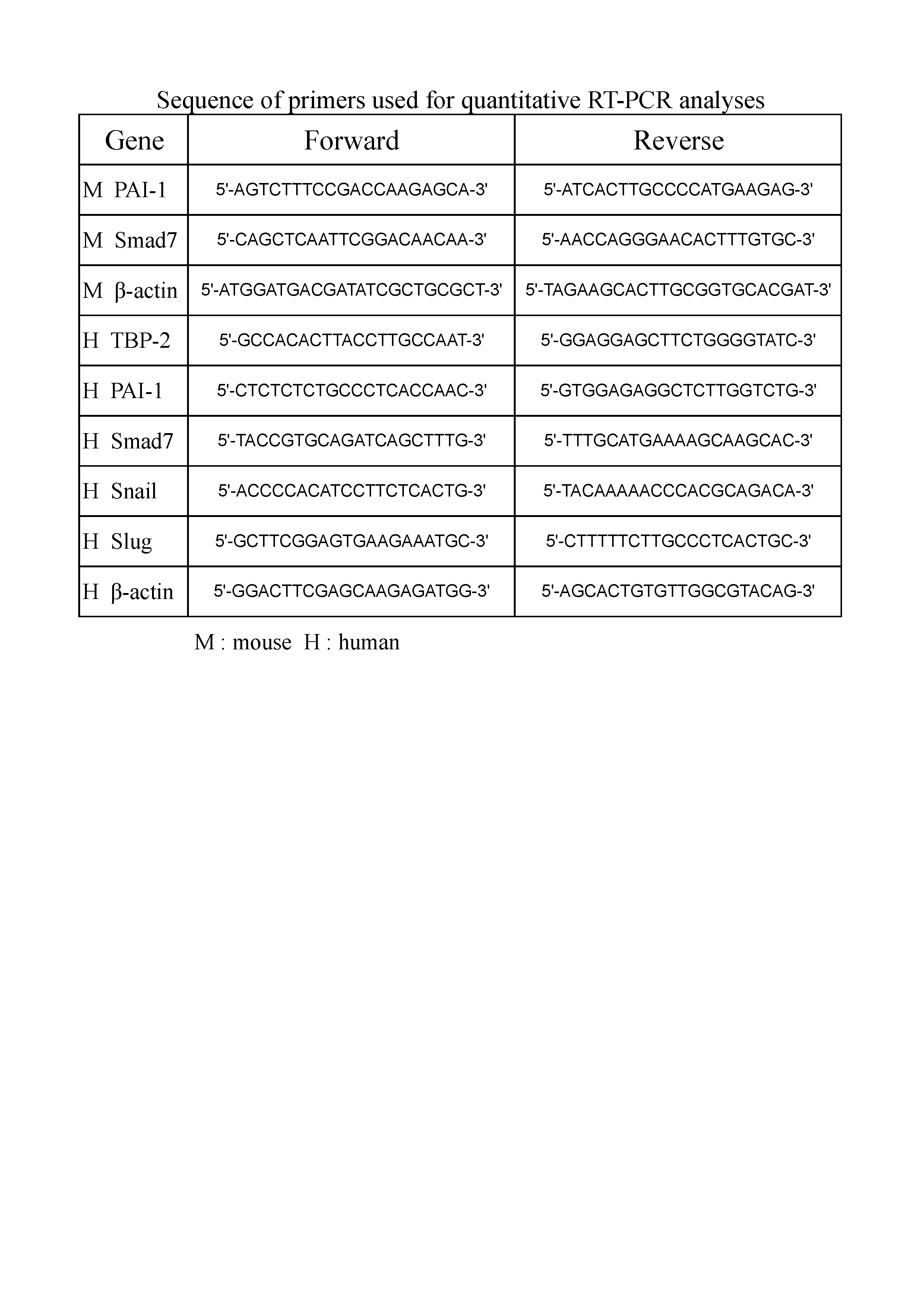

Supplement: Table S1 — Primer sequences for real-time PCR analyses. (TIFF) [file pone.0039900.s002.tif]
